# Longitudinal multi-night home-based sleep assessment before and after metabolic bariatric surgery: protocol for the SLEEP-BAM cohort

**DOI:** 10.1093/sleepadvances/zpag109

**Published:** 2026-10-09

**Authors:** Ephrahim Edoja Jerry, R A Bouwman, Simon W Nienhuijs

**Affiliations:** Department of Surgery, Catharina Hospital Eindhoven, Michelangelolaan 2, 5623 EJ Eindhoven, The Netherlands; Department of Electrical Engineering, Signal Processing Systems, Eindhoven University of Technology, Eindhoven 5612 AE, The Netherlands; Department of Electrical Engineering, Signal Processing Systems, Eindhoven University of Technology, Eindhoven 5612 AE, The Netherlands; Department of Anesthesiology, Catharina Hospital Eindhoven, Michelangelolaan 2, 5623 EJ Eindhoven, The Netherlands; Department of Surgery, Catharina Hospital Eindhoven, Michelangelolaan 2, 5623 EJ Eindhoven, The Netherlands; NUTRIM School of Nutrition and Translational Research in Metabolism, Maastricht University, Maastricht, The Netherlands

**Keywords:** sleep, obesity, bariatric surgery, metabolic surgery, wearable electronic devices, photoplethysmography, actigraphy, longitudinal studies, sleep quality, circadian rhythm

## Abstract

**Study Objectives:**

Sleep disturbances are common in people with obesity and in candidates for metabolic bariatric surgery, but sleep is often evaluated mainly through obstructive sleep apnea screening or subjective questionnaires. SLEEP-BAM aims to evaluate longitudinal changes in sleep quality from before surgery to approximately 1 year after surgery using multi-night home-based wearable monitoring. The primary objective is to assess change in Garmin Sleep Score, a pragmatic device-derived composite measure of sleep quality.

**Materials and Methods:**

This single-center, prospective, within-subject cohort study will include 50 adults scheduled for primary metabolic bariatric surgery at Catharina Hospital Eindhoven, The Netherlands. Participants will complete two 5-night home measurement periods: between 3 months and 2 weeks preoperatively and approximately 1 year postoperatively. During each period, participants will wear a Garmin Index Sleep Monitor and a research-grade upper-arm wearable and will complete sleep diaries and validated questionnaires. Objective sleep, sleep timing, peripheral oxygen saturation, photoplethysmography-derived pulse rate variability features, subjective sleep, quality of life, and clinical outcomes including percentage total weight loss will be assessed.

**Results:**

Recruitment commenced in September 2026. Data collection is ongoing, and no participant outcome data have yet been analyzed.

**Conclusions:**

SLEEP-BAM will characterize sleep trajectories after metabolic bariatric surgery using repeated real-world sleep assessment. The findings may clarify how objective and subjective sleep measures change after major weight loss and support future sleep-focused interventions or wearable-derived sleep algorithms in this population.

**Clinical trial registration:**

ClinicalTrials.gov NCT07693491

**Protocol information:**

SLEEP-BAM; protocol ID NL-011757; MEC-U registration number R26.043; protocol version 1.2, July 2, 2026.

Clinical Trial Information.

**Name:**

Longitudinal multi-night home-based sleep assessment before and after metabolic bariatric surgery: protocol for the SLEEP-BAM cohort

URL: https://clinicaltrials.gov/study/NCT07693491

## Teaser

**Statement of Significance** Sleep problems are common in people undergoing metabolic bariatric surgery, yet routine care generally focuses on obstructive sleep apnea and may overlook sleep quality, timing, and regularity. SLEEP-BAM will use repeated home-based measurements to characterize sleep before and after surgery in the same participants. Combining sleep diaries and validated questionnaires with consumer-grade and physiological wearable data may help bridge the gap between clinical sleep symptoms and real-world objective sleep patterns. This protocol provides a reproducible framework for studying sleep as a multidimensional outcome after major weight loss and may inform future sleep-focused care pathways and wearable-derived sleep research.

## Graphical abstract

**Graphical abstract:**
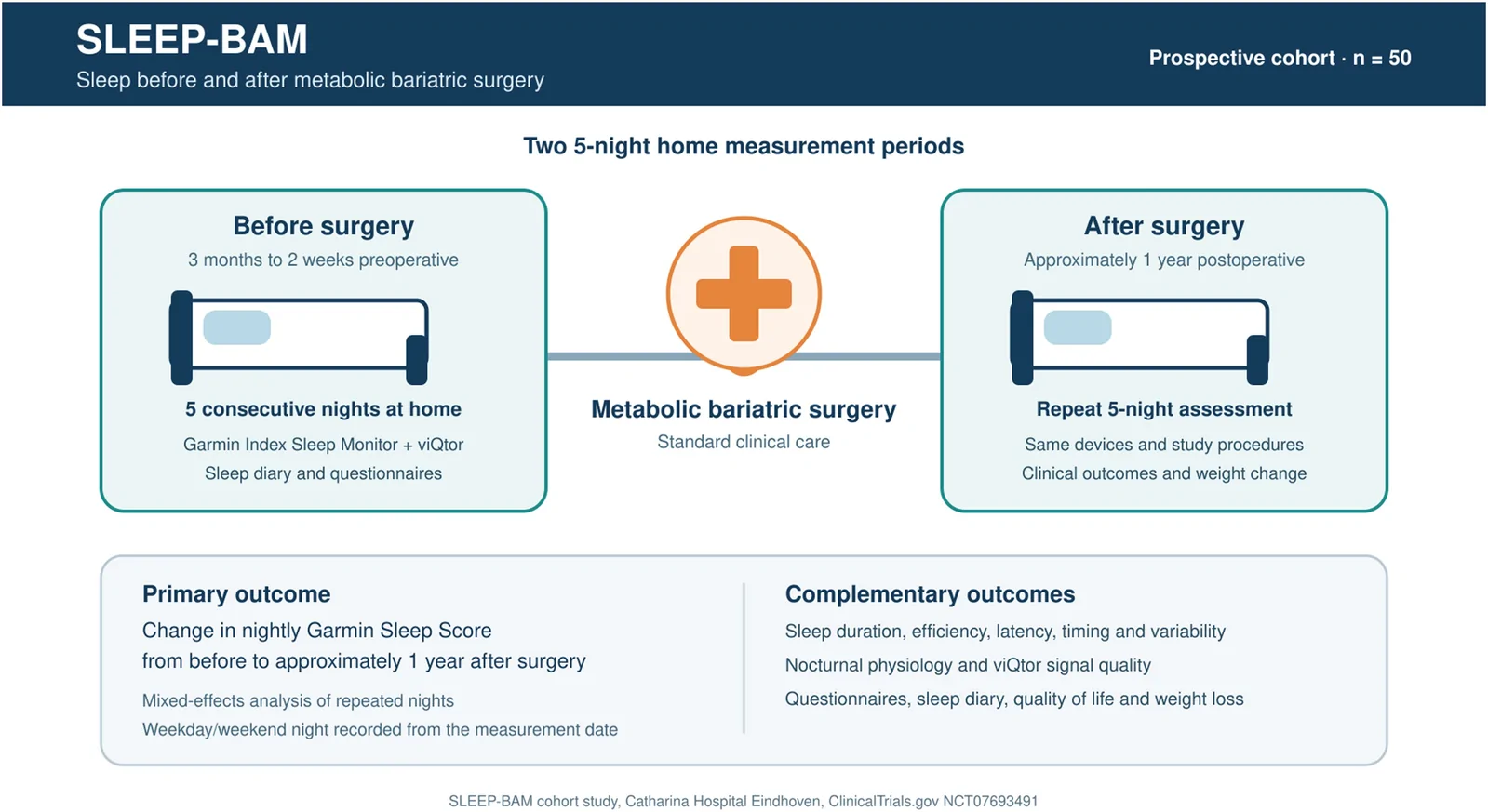

## Introduction

### Background

Obesity is a chronic, multifactorial condition influenced by behavioral, psychological, genetic, circadian, and sleep-related factors. Insufficient sleep and circadian misalignment are associated with adverse metabolic health and obesity [1]. Metabolic bariatric surgery is an effective treatment for severe obesity and related comorbidities, but sleep assessment within routine pathways is often centered on obstructive sleep apnea.

Sleep disturbance, insomnia symptoms, and poor subjective and objective sleep quality are common among people seeking metabolic bariatric surgery [2, 3]. Prospective evidence suggests that sleep quality can improve after surgery [3], while pilot data suggest that longer sleep duration and better sleep quality may be associated with greater immediate and sustained postoperative weight loss [4]. The existing evidence is limited by frequent reliance on subjective questionnaires, cross-sectional comparisons, or single-night measurements. Such approaches may not adequately represent night-to-night variability or sleep timing under real-world conditions.

Longitudinal multi-night home monitoring can complement subjective measures while limiting participant burden. Consumer devices provide repeatable, pragmatic sleep estimates, whereas research-grade physiological wearables can provide raw photoplethysmography and accelerometry signals. Together, these devices provide complementary objective data: Garmin provides consumer-derived sleep estimates, whereas viQtor provides research-accessible physiological and motion signals. These objective measures will be interpreted alongside subjective sleep diaries and validated questionnaires, while remaining distinct from diagnostic sleep testing.

### Rationale

SLEEP-BAM addresses the absence of longitudinal, multi-night sleep assessment before and after metabolic bariatric surgery. Its within-participant design reduces between-person variability, while five consecutive nights at each time point provide a more representative estimate of habitual sleep and night-to-night variability than a single-night assessment, while limiting participant burden. The postoperative assessment at approximately 1 year coincides with substantial weight loss and routine clinical follow-up. For this study, substantial weight loss is operationally defined as at least 25% total weight loss, calculated as 100 × (preoperative weight − postoperative weight) / preoperative weight. The Garmin Sleep Score was selected as a pragmatic primary outcome because it provides a single nightly summary that can be collected repeatedly with low participant burden. However, the score is generated by a proprietary algorithm, and the exact components and their relative weighting are not publicly available. It is therefore treated as a non-diagnostic longitudinal measure rather than a substitute for polysomnography. Changes in the Sleep Score will be interpreted alongside individual Garmin-derived sleep variables, sleep diaries, validated questionnaires, and complementary viQtor measurements. The viQtor device is included to complement Garmin’s proprietary sleep estimates with research-accessible physiological and motion signals.

### Objectives and hypotheses

The primary objective is to estimate the within-participant change in Garmin Sleep Score from the preoperative measurement period to approximately 1 year after surgery. The primary hypothesis is that the mean Garmin Sleep Score will be higher postoperatively.

Secondary objectives are to estimate changes in objective sleep duration, efficiency, latency, timing, night-to-night variability, nocturnal pulse rate, respiratory rate, peripheral oxygen saturation, skin temperature, movement, signal quality, photoplethysmography-derived pulse rate variability, subjective sleep, daytime sleepiness, anxiety and depression symptoms, and obesity-specific quality of life; to quantify agreement between directly comparable diary and wearable measures; to explore associations between sleep change and postoperative weight loss; and to assess exploratory viQtor-derived features, including their associations with temporally corresponding Garmin-derived sleep and physiological measures. Secondary and device-development analyses are hypothesis-generating.

## Materials and methods

### Study design and setting

SLEEP-BAM is a prospective, single-center, within-participant longitudinal cohort study at Catharina Hospital Eindhoven, The Netherlands. The study does not assign a clinical intervention or comparator; metabolic bariatric surgery is delivered as standard care independently of study participation. The protocol is reported in accordance with applicable Standard Protocol Items: Recommendations for Interventional Trials (SPIRIT) 2013 items and includes a participant timeline (Figure 1) [5].

**Figure 1:**
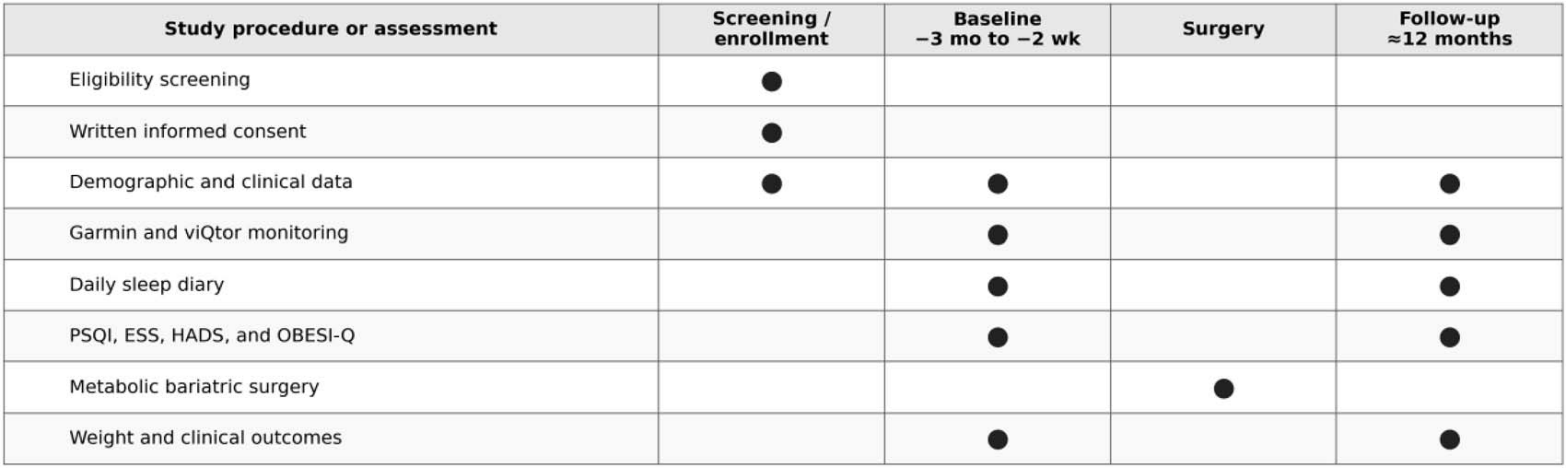
SPIRIT schedule of enrollment, standard-care exposure, and assessments for the SLEEP-BAM cohort study. Filled circles indicate the planned timing of each procedure or assessment. ESS, Epworth Sleepiness Scale; HADS, Hospital Anxiety and Depression Scale; PSQI, Pittsburgh Sleep Quality Index. Participant timeline showing screening and informed consent, baseline assessments and 5-night home monitoring before surgery, metabolic bariatric surgery as standard care, and repeated assessments and 5-night home monitoring approximately 1 year after surgery.

Each participant will complete two home measurement periods of 5 consecutive nights: a baseline period between 3 months and 2 weeks before surgery and a postoperative period approximately 1 year after surgery. The measurement periods may include both weekday and weekend nights. Each night will be classified according to the date on which the participant attempts sleep, with Friday and Saturday nights classified as weekend nights and Sunday through Thursday nights as weekday nights. Study procedures will coincide with routine outpatient visits where feasible.

### Study population

Adults scheduled for primary Roux-en-Y gastric bypass, sleeve gastrectomy, or one-anastomosis gastric bypass within standard care will be eligible for screening. Revisional procedures are excluded, the full inclusion and exclusion criteria are presented in Table 1.

**Table 1:** Eligibility criteria

Inclusion criteria	Exclusion criteria
Age ≥ 18 years.	Revisional bariatric surgery.
Body mass index ≥40 kg/m^2^, or ≥ 35 kg/m^2^ with obesity-related complications.	Shift work or irregular night work expected to interfere with sleep measurement.
Scheduled for primary metabolic bariatric surgery.	Diagnosed obstructive sleep apnea currently treated with continuous or bilevel positive airway pressure.
Able and willing to wear both devices for 5 consecutive nights at both time points and complete diaries and questionnaires.	High clinical suspicion of obstructive sleep apnea during routine work-up requiring further diagnostic evaluation or treatment.
Able to provide written informed consent.	Chronic use at the time of enrollment of benzodiazepines, zolpidem, or another sedative or sleep aid judged by the treating physician to substantially affect habitual sleep or the study measurements.
	Inability to wear the devices or complete study procedures.

As part of the routine preoperative work-up, a STOP-BANG score of 5–8 will be considered indicative of a high risk of obstructive sleep apnea. The STOP-BANG score alone will not result in exclusion. Participants will only be excluded when the clinical assessment leads to referral for further diagnostic evaluation or initiation of treatment before completion of the baseline measurement period.

### Recruitment and informed consent

Potential participants will be identified within the standard multidisciplinary metabolic surgery pathway. A researcher will introduce the study during the first nurse-led preoperative group session. Patients will receive written information and at least 1 week to consider participation. An individual conversation with the researcher will be offered before consent to address procedures, burden, data handling, voluntary participation, and withdrawal. Written informed consent will be obtained before any study-specific procedure. Declining or withdrawing will not affect clinical care.

### Participant timeline

The baseline interval was selected to measure habitual preoperative sleep before immediate perioperative influences where feasible. Follow-up at approximately 1 year corresponds to a clinically relevant phase of postoperative weight loss. The exact interval from surgery to each measurement period will be recorded.

### Study procedures

Study procedures will be completed in the following order:

(1) Screening and consent. The research team will confirm eligibility, provide study information, answer questions, and obtain written informed consent.
(2) Baseline preparation. Between 3 months and 2 weeks before surgery, the team will record demographic and available clinical data; administer the Pittsburgh Sleep Quality Index (PSQI), Epworth Sleepiness Scale (ESS), Hospital Anxiety and Depression Scale (HADS), and OBESI-Q; and fit both devices. A trained member of the research team will provide standardized verbal and written instructions using a study-specific instruction sheet. Participants will be shown how to position and secure each device, how to start and maintain device use, and how to recognize improper fit or device malfunction. They will be instructed to wear both devices continuously during the evening and overnight for 5 consecutive nights, to avoid exchanging devices or changing study settings, and to document bedtime, attempted sleep time, awakenings, final wake time, device removal, naps, and any technical problems in the sleep diary. Participants will be asked to contact the research team if a device becomes uncomfortable, is removed unintentionally, or appears not to function correctly. Participants will not be given access to the Garmin application, study account, Sleep Score, or other Garmin-derived results during either measurement period. Garmin results will only be accessed by the research team after return and synchronization of the device.
(3) Baseline home measurement. Participants will wear both devices for 5 consecutive nights, applying them in the evening several hours before their intended bedtime and keeping them in place until after final awakening the following morning. The sleep diary will be completed each morning. The diary records bedtime, attempted sleep time, estimated sleep onset, awakenings, final wake time, device removal, naps, and subjective sleep quality.
(4) Return and data check. Participants will return the devices, diary, and questionnaires. The team will download data using the designated software or secure offline procedures, match files by study code, document device use and technical problems, and apply the predefined validity rules without reference to postoperative outcomes.
(5) Postoperative follow-up. Approximately 1 year after surgery, the questionnaires, diary, and 5-night wearable procedure will be repeated. Weight, surgery type, relevant comorbidities, medication use, and available clinical outcomes will be abstracted from the medical record according to the approved protocol. Medication changes occurring after enrollment that may affect sleep, including initiation, discontinuation, or dose adjustment of sedatives, hypnotics, antidepressants, or other sleep-modifying medication, will be recorded. Such changes will not automatically result in participant exclusion but will be considered in the interpretation and analysis of postoperative sleep outcomes.

### Measures and devices

#### Garmin Index Sleep Monitor

An overview of the study measures, data sources, and assessment timing is provided in Table 2. The Garmin Index Sleep Monitor is a commercially available upper-arm wearable that estimates sleep from accelerometry and photoplethysmography. Previous validation studies of earlier Garmin devices indicate that consumer wearables can provide useful repeated sleep estimates, although performance varies across sleep variables and device generations [14–17]. No independent model-specific validation of the Garmin Index Sleep Monitor is currently available, and findings from earlier Garmin models cannot be directly extrapolated to this device. Its composite Sleep Score is used as the primary longitudinal outcome, while the individual sleep components are evaluated as secondary outcomes. Study outputs include the Garmin Sleep Score and device-derived sleep duration, efficiency, latency, stages, awakenings, and timing measures. These proprietary outputs will be used as pragmatic repeated research measures and will not be interpreted as diagnostic or polysomnographic measurements. The device model, serial number, firmware version, Garmin software version, and any relevant updates will be recorded for each measurement period. Because software and firmware may be updated during the study, the version active during each measurement period will be retained in the study records [18].

**Table 2:** Study measures, sources, and timing

Domain	Measure or variable	Source	Timing
Primary sleep outcome	Garmin Sleep Score (0–100)	Garmin Index Sleep Monitor	Each valid night in both 5-night periods
Objective sleep	Total sleep time, sleep efficiency, sleep latency, sleep stages, nocturnal awakenings, bedtime, wake time, mid-sleep time, and night-to-night variability	Garmin device outputs	Each valid night in both periods
Nocturnal physiology	Pulse rate, respiratory rate, peripheral oxygen saturation, skin temperature, motion, signal quality, and exploratory photoplethysmography-derived pulse rate variability features, including RMSSD*, SDNN*, and, where signal quality and recording duration permit, frequency-domain measures such as low-frequency and high-frequency power.	viQtor device	Each usable recording in both periods
Subjective sleep	PSQI [6], ESS [7], and daily sleep diary	Participant report	Questionnaires at both periods; diary daily
Mood and quality of life	HADS [8] and OBESI-Q [9]	Participant report	Both periods
Clinical outcomes	Weight, body mass index, percentage total weight loss, surgery type, comorbidities, medication use, and available obstructive sleep apnea status	Clinical record	Baseline and follow-up, as available

^*^Root mean square of successive differences (RMSSD) and standard deviation of normal-to-normal intervals (SDNN).

#### viQtor device

The viQtor upper-arm wearable (SmartQare, Eindhoven, The Netherlands) records photoplethysmography and tri-axial accelerometry and provides physiological signals relevant to sleep research. Its vital-sign monitoring performance has been evaluated in postoperative noncardiac surgery patients [10], but its exploratory sleep features in the present population are not diagnostic. Data will be stored locally on the integrated SD card and read by the research team using secure offline procedures.

The two devices serve complementary purposes. The Garmin device provides proprietary, consumer-oriented nightly sleep estimates and a composite Sleep Score, whereas viQtor provides access to underlying physiological and motion signals for signal-quality assessment and exploratory feature derivation. Because both devices will be worn simultaneously, temporally corresponding outputs will be compared exploratorily to assess concordance, systematic differences, and the feasibility of deriving sleep-relevant measures from the viQtor signals. These analyses are not intended as formal validation against polysomnography.

### Data-quality and validity rules

For the primary outcome, a valid Garmin night requires an available nightly Garmin Sleep Score and at least 4 hours of usable recording overlapping the sleep period indicated by the diary and/or device. This threshold was selected to ensure that a substantial proportion of the principal sleep episode was captured while minimizing unnecessary exclusion of otherwise usable recordings. Recording during evening wakefulness alone is insufficient. The primary measurement period is considered valid when at least 3 valid Garmin nights are available. Garmin nights failing these rules will be excluded from the primary outcome analysis and the reason will be logged.

Night-to-night variability will be calculated for continuous sleep variables as the within-participant standard deviation across valid nights within each measurement period. Variability will only be calculated when at least 3 valid nights are available. For bedtime, wake time, and mid-sleep time, clock times will first be converted to minutes relative to noon so that times occurring across midnight remain chronologically ordered.

viQtor analyses will use device-specific usable-signal criteria and will not determine whether a Garmin night is valid. Signal loss, nonwear, implausible timestamps, and technical failure will be documented separately. Plausible extreme values will be retained; only values attributable to a documented technical or data-processing error will be removed. All data-quality decisions will be made using predefined rules before the outcome comparison is examined.

### Outcomes

#### Primary outcome

The primary outcome is the within-participant change in Garmin Sleep Score (range, 0–100) from the preoperative measurement period to approximately 1 year after surgery. The primary analysis will estimate the mean difference between the two measurement periods using a linear mixed-effects model that includes all available valid nightly scores. As a sensitivity analysis, participant-level mean scores will be compared among participants with at least 3 valid nights in both measurement periods.

#### Secondary outcomes

Secondary outcomes are changes in Garmin-derived sleep duration, efficiency, latency, stages, awakenings, bedtime, wake time, mid-sleep time, and within-period variability; viQtor-derived nocturnal physiology, signal-quality measures, and exploratory photoplethysmography (PPG)-derived pulse rate variability features including the root mean square of successive differences (RMSSD) and standard deviation of normal-to-normal intervals (SDNN); PSQI, ESS, HADS, OBESI-Q, and diary outcomes; and weight, body mass index, and percentage total weight loss. Agreement analyses are limited to directly comparable diary and wearable variables. Exploratory analyses will examine associations between sleep change and clinical outcomes and will compare temporally corresponding Garmin- and viQtor-derived measures. These technical analyses will assess correlation, agreement where measures are sufficiently comparable, systematic differences, signal availability, and the feasibility of deriving sleep-relevant physiological features from viQtor data. No formal diagnostic-accuracy or device-validation claim is planned.

### Sample size

The target sample size is 50 participants. The sample size calculation is based on detecting a within-subject change in Garmin Sleep Score between baseline and 1 year postoperatively. Assuming a moderate within-subject effect size of Cohen *d* = 0.50, a two-sided alpha of .05, and 80% power, 34 participants with complete paired measurements are required [11, 12]. This paired-means calculation is used as a pragmatic design approximation because reliable study-specific variance components for a formal sample size calculation based on nightly mixed-effects modeling were not available before recruitment. The linear mixed-effects model remains the primary analysis, while the participant-level paired analysis will be performed as a sensitivity analysis.

Because wearable-based longitudinal studies are prone to incomplete recordings, insufficient valid nights, device nonadherence, signal-quality issues, and loss to follow-up, the target sample size was increased from 34 participants with complete paired measurements to 50 enrolled participants. This corresponds to an allowance of approximately 32% for incomplete paired datasets. No precise study-specific estimate of missing nightly recordings was available before recruitment; therefore, the inflation was selected as a pragmatic safeguard against both technical data loss and participant attrition. No routine replacement of withdrawn participants is planned because this target already accounts for expected incomplete datasets.

### Analytic strategies

Analyses will follow a prespecified statistical analysis plan finalized before the postoperative outcome comparison. Baseline characteristics, feasibility, adherence, valid-night counts, and device failures will be summarized using means and standard deviations or medians and interquartile ranges, as appropriate, and counts with percentages. Model assumptions will be assessed using residual diagnostics. All tests will be two-sided. The software, packages, and version numbers used for each analysis will be specified in the statistical analysis plan and reported in the final results manuscript.

#### Primary analysis

Nightly Garmin Sleep Score will be analyzed with a linear mixed-effects model containing measurement period (preoperative vs postoperative) and night type (weekday vs weekend) as fixed effects and participant as a random intercept. The model uses all available valid nightly scores and accounts for repeated nights within participants. The fixed-effect contrast for measurement period will be reported as an adjusted mean difference with 95% confidence interval and *p* value. A standardized within-participant effect size (Cohen *d*_z) with 95% confidence interval will also be reported. If a documented Garmin firmware, software, or scoring-algorithm update occurs during data collection, a version or update indicator will be included when identifiable and statistically estimable. Where feasible, sensitivity analyses will restrict or stratify measurements according to the recorded device or software version.

Sensitivity analyses will compare participant-level period means among those with at least 3 valid nights in both periods using a paired *t* test or Wilcoxon signed-rank test if the distribution of differences is markedly nonnormal. A further sensitivity analysis will include the exact postoperative interval and measurement season if these vary sufficiently and the model remains stable.

#### Secondary and exploratory analyses

Continuous secondary outcomes will be analyzed with analogous mixed models or paired analyses, depending on the measurement structure. Night type will be included as a fixed effect in models of nightly outcomes. Results will include raw change estimates, 95% confidence intervals, and a standardized effect size appropriate to the analysis. Directly comparable diary and wearable measures, such as total sleep time and sleep timing, will be evaluated using Bland–Altman plots and limits of agreement in addition to Pearson or Spearman correlations. Correlation alone will not be interpreted as agreement.

Associations between change in sleep and percentage total weight loss, body mass index change, and quality-of-life change will be explored using correlation and regression models. Potential confounders, including age, sex, baseline body mass index, surgery type, relevant comorbidities, medication changes affecting sleep, measurement season, and the exact interval between surgery and postoperative assessment, will be considered where clinically relevant and statistically feasible.

Exploratory interaction analyses will only be performed if the sample size and cell counts support stable estimation. Medication changes affecting sleep will be summarized descriptively and, where the number of affected participants permits, evaluated through covariate adjustment or sensitivity analyses excluding measurements obtained during relevant medication use. No formal multiplicity adjustment is planned for secondary and exploratory analyses; interpretation will emphasize effect estimates, uncertainty, and hypothesis generation rather than isolated *p* values.

#### Missing data and outliers

The frequency and reasons for missing data will be reported separately for participant attrition, missing measurement periods, insufficient valid nights, device nonwear, signal loss, and technical failure. Because no directly applicable prior estimate was available for the combination of devices and study procedures used in SLEEP-BAM, no fixed percentage of missing nightly recordings was assumed. The protocol allows up to 2 missing or invalid nights within each planned 5-night period for period-level sensitivity analyses requiring at least 3 valid nights.

The mixed model provides valid likelihood-based inference under a missing-at-random assumption conditional on included information; outcome values will not be imputed for the primary analysis. Questionnaire items will be scored according to instrument-specific rules, without an additional arbitrary percentage threshold. Missing covariates may be multiply imputed if the extent and pattern warrant this and the imputation model is defensible. Participants with and without postoperative data will be compared descriptively. A complete-period analysis and, if informative, a pattern-based sensitivity analysis will assess robustness to attrition. Documented technical errors will be excluded; plausible extreme observations will remain, with influence assessed in sensitivity analyses.

### Data management and privacy

Participants will be assigned a unique study code. The reidentification key will be stored separately in an access-restricted, password-protected location. Study data will be stored on secure hospital systems and managed in ResearchManager, a Good Clinical Practice-compliant electronic data-capture system certified to ISO 27001 and NEN 7510. Access will be role-based and limited to authorized personnel. Data will be handled in accordance with the General Data Protection Regulation and Dutch law.

A limited pseudonymized dataset containing Garmin- and viQtor-derived sleep data may be transferred to SmartQare under the applicable data-sharing agreement for signal-quality assessment and exploratory feature development. No names, hospital numbers, questionnaire responses, clinical outcomes, or reidentification key will be transferred. Derived features may be returned to the research team. Garmin software will be used for data extraction where technically required; the research team will not share medical-record data, hospital identifiers, questionnaires, or directly identifying clinical data with Garmin.

No participant-level dataset is available at the protocol stage. After completion, deidentified data may be shared by the corresponding author upon reasonable request, subject to institutional approval, participant privacy, applicable law, and data-sharing agreements. The final manuscript will describe any available code or analytic materials and the conditions for access.

## Ethical Considerations and Trial Registration

The study will be conducted in accordance with the Declaration of Helsinki, Good Clinical Practice principles, applicable Dutch legislation including the Medical Research Involving Human Subjects Act, and relevant medical device regulations. The study has been approved by Medical Research Ethics Committees United (MEC-U; registration number R26.043; NL-number NL-011757/case R038704; approval date July 3, 2026). Written informed consent will be obtained from all participants before study procedures.

The study is observational and low risk. Participants will undergo metabolic bariatric surgery as part of routine clinical care independent of study participation. Study burden consists of wearing two noninvasive upper-arm devices for 5 consecutive nights at two time points and completing questionnaires and sleep diaries. Potential risks are limited to mild skin irritation, local discomfort, pressure marks, inconvenience, or altered behavior due to awareness of being monitored. No pharmacological intervention, radiation exposure, or additional surgical intervention is involved. Individual sleep results will not be used to guide clinical decisions during the study.

The study has been registered at ClinicalTrials.gov (NCT07693491).

## Monitoring and Quality Assurance

A risk-based monitoring approach will be applied according to Catharina Hospital Research & Innovation Department monitoring policy and Good Clinical Practice. Monitoring will include verification of informed consent procedures, protocol adherence, data completeness and accuracy, and secure data handling. At least one initiation visit and one close-out visit will take place, with additional remote checks if needed.

## Study Status

At the time of manuscript preparation, this is a study protocol and no participant outcome data have been analyzed. Recruitment commenced in September 2026 and is expected to continue for approximately 12 months. Total study duration is estimated at approximately 24 months, including recruitment, 12-month postoperative follow-up, data analysis, and dissemination.

## Discussion

### Expected contributions

SLEEP-BAM is designed to provide longitudinal, multi-night, home-based insight into sleep quality before and after metabolic bariatric surgery. The study addresses a clinically relevant gap in metabolic surgery research by moving beyond single-night or purely subjective sleep assessment and by evaluating sleep as a multidimensional construct that includes quality, duration, efficiency, timing, regularity, and physiological parameters.

A key strength of the study is its within-subject design, which reduces interindividual variability and allows each participant to serve as their own control. The timing of postoperative follow-up at approximately 1 year aligns with a clinically meaningful period of substantial postoperative weight loss and routine follow-up. The use of both sleep diaries and validated questionnaires will help contextualize device-derived data and assess the degree of agreement between subjective and objective sleep measures.

The dual-device approach is another strength. The Garmin device provides pragmatic consumer-grade sleep outputs that are accessible and scalable, while the research version of the viQtor device provides physiological signals and raw photoplethysmography data with potential value for algorithm development. A CE-marked medical-device version of viQtor is available for continuous vital-sign monitoring, which may provide a pathway for future clinical translation if sleep-related algorithms are successfully developed and validated [10]. However, the research device used in this study is not CE-marked, and the exploratory sleep-related features evaluated here are not intended for clinical decision-making. This combination allows both clinical sleep trajectory evaluation and exploratory technical analysis of sensor-derived features in a population with obesity undergoing major weight loss.

The simultaneous use of both devices also permits an exploratory technical comparison between readily available proprietary sleep outputs and research-accessible physiological signals. This may provide insight into which measures are concordant across devices, where systematic differences occur, and whether viQtor data can support future development of sleep-related features. These comparisons will remain exploratory because neither device is evaluated against polysomnography in the present study.

### Limitations

The study has limitations. The Garmin Sleep Score is generated by a proprietary consumer-grade algorithm, and its exact formula and the relative weighting of its components are not publicly available. It is not equivalent to polysomnography and will therefore be interpreted as a pragmatic longitudinal measure rather than a diagnostic gold standard. Independent validation specifically for the Garmin Index Sleep Monitor is currently unavailable, and validation findings from earlier Garmin models may not generalize to this device. Firmware, software, or scoring-algorithm updates during the study could introduce systematic differences between the preoperative and postoperative measurements. Recording the active versions and performing sensitivity analyses where feasible may help evaluate, but cannot eliminate, this risk. The study is single center and observational, which may limit generalizability and preclude causal inference. The sample size was based on a pragmatic paired-means approximation and may limit subgroup analyses and exploratory modeling.

Selection bias may occur because participants willing to wear devices and complete diaries may differ from nonparticipants. The Hawthorne effect may alter sleep behavior during measurement periods [13]. Selective loss to follow-up may occur if participants with poorer postoperative outcomes, worse sleep, or lower motivation are less likely to complete the 12-month measurement. Although patients with treated obstructive sleep apnea or high clinical suspicion requiring further evaluation will be excluded, residual undiagnosed or mild sleep-disordered breathing cannot be ruled out. Finally, substantial postoperative weight loss may influence device fit and signal quality, which will be addressed through standardized instructions, device fit checks, and predefined data-quality criteria.

## Conclusions

The SLEEP-BAM study will evaluate changes in sleep quality before and after metabolic bariatric surgery using longitudinal, multi-night, home-based wearable monitoring combined with validated subjective measures. Findings may clarify how sleep evolves during the first postoperative year, how subjective and objective sleep measures relate to each other, and whether sleep assessment should be integrated into metabolic surgery care pathways. Exploratory viQtor photoplethysmography analyses may also inform future development of sleep-related algorithms for wearable monitoring in surgical populations.

## Supplementary Material

SLEEP_BAM_SPIRIT_2013_Checklist_zpag109

## Data Availability

No participant data are available at the protocol stage. After study completion, deidentified data may be made available from the corresponding author upon reasonable request and subject to institutional approval, participant privacy, General Data Protection Regulation requirements, and applicable data-sharing agreements.
