# Supplementary material for: Longitudinal multi-night home-based sleep assessment before and after metabolic bariatric surgery: protocol for the SLEEP-BAM cohort
Source: Sleep Adv. 2026 Oct 9;7(4):zpag109. doi: 10.1093/sleepadvances/zpag109 (PMC13744282; doi:10.1093/sleepadvances/zpag109)
Supplement: Supplementary file 1 [file sleep_bam_spirit_2013_checklist_zpag109.docx]

**SPIRIT 2013 Checklist - SLEEP-BAM Protocol Paper**

*Longitudinal Multi-Night Home-Based Sleep Assessment Before and After Metabolic Bariatric Surgery: Protocol for the SLEEP-BAM Cohort Study*

**Article type:** Protocol Paper. **Journal:** SLEEP Advances. **Trial registration:** ClinicalTrials.gov NCT07693491. **Ethics approval:** Medical Research Ethics Committees United (MEC-U), R26.043, 3 July 2026.

**Note:** SPIRIT was developed for interventional trials. This protocol describes a prospective observational cohort study; items that are specific to randomized allocation, blinding, treatment discontinuation, investigational products, and trial steering committees are marked not applicable with a justification. Locations refer to the revised SLEEP Advances manuscript. Section headings are used because pagination may differ between the tracked-changes and clean files.

| **Section** | **Item** | **SPIRIT checklist item** | **Location in revised manuscript** | **Comments / justification** |
| --- | --- | --- | --- | --- |
| Administrative information | 1 | Title. Descriptive title identifying the study design, population, interventions or exposures, and, if applicable, trial acronym. | Title page | Title includes Protocol, prospective cohort design, metabolic bariatric surgery, multi-night home-based sleep assessment, and SLEEP-BAM acronym. |
| Administrative information | 2a | Trial registration. Trial identifier and registry name. If not yet registered, name of intended registry. | Title page; Abstract; Study Status | ClinicalTrials.gov NCT07693491 is listed. |
| Administrative information | 2b | Protocol version. All items from the WHO Trial Registration Data Set. | Protocol Information; ClinicalTrials.gov registration | Protocol version, registry information, and ethics details are stated; WHO data-set elements are recorded in ClinicalTrials.gov. |
| Administrative information | 3 | Protocol version. Date and version identifier. | Protocol Information | Protocol version 1.2 and date 02 July 2026 are stated. |
| Administrative information | 4 | Funding. Sources and types of financial, material, and other support. | Funding | SmartQare provided study devices; no role in study design, data collection, analysis, interpretation, or writing is stated. |
| Administrative information | 5a | Roles and responsibilities: contributors. Names, affiliations, and roles of protocol contributors. | Title page; Author Contributions | Authors, affiliations, corresponding author, and contributions are stated. |
| Administrative information | 5b | Roles and responsibilities: sponsor and funder. Name and contact information for trial sponsor. | Title page; Funding; ClinicalTrials.gov registration | Catharina Hospital Eindhoven is the sponsor; SmartQare provides devices and technical support. |
| Administrative information | 5c | Roles and responsibilities: committees. Role of study sponsor and funders in design, data collection, management, analysis, interpretation, report writing, and publication. | Funding; Data Management and Privacy | Funder role and data handling with SmartQare are described. |
| Administrative information | 5d | Roles and responsibilities: coordinating centers and committees. Composition, roles, and responsibilities of coordinating center, steering committee, endpoint adjudication committee, data management team, and other groups. | Monitoring and Quality Assurance; Author Contributions | Risk-based monitoring is described. No formal steering committee, endpoint adjudication committee, or DSMB is planned; study is single-center and low risk. |
| Introduction | 6a | Background and rationale. Description of research question and justification for undertaking the study. | Introduction and Rationale; Rationale | The revised rationale explains the need for repeated home monitoring, the 1-year follow-up, and the pragmatic role and limitations of the Garmin Sleep Score. |
| Introduction | 6b | Choice of comparators. Explanation for choice of comparators. | Rationale; Objectives and Hypotheses; Study Design and Setting | No external comparator group; within-subject pre-post comparison is justified to reduce interindividual variability. |
| Introduction | 7 | Objectives. Specific objectives or hypotheses. | Objectives and Hypotheses | Primary objective and secondary objectives/hypotheses are stated. |
| Methods: participants, interventions, outcomes | 8 | Study design. Description of study design, including type, allocation ratio, and framework. | Study Design and Setting; Figure 1 | Prospective single-center within-subject longitudinal cohort with baseline and 1-year follow-up measurements. |
| Methods: participants, interventions, outcomes | 9 | Study setting. Description of study settings and locations. | Study Design and Setting | Catharina Hospital Eindhoven Obesity Center, the Netherlands. |
| Methods: participants, interventions, outcomes | 10 | Eligibility criteria. Inclusion and exclusion criteria for participants and study centers. | Study Population; Table 1 | Eligibility criteria include treated OSA and clinically suspected OSA. STOP-BANG scores of 5-8 indicate high risk, while exclusion depends on the clinical referral or treatment pathway rather than the score alone. |
| Methods: participants, interventions, outcomes | 11a | Interventions/exposures. Interventions for each group with sufficient detail for replication. | Study Procedures; Measures and Devices | Observational exposure is routine metabolic bariatric surgery; study procedures are wearable monitoring, sleep diary, questionnaires, and extraction of clinical data. |
| Methods: participants, interventions, outcomes | 11b | Criteria for discontinuing or modifying interventions. Criteria for discontinuing or modifying allocated interventions. | N/A | Not applicable: no allocated intervention or study treatment. Withdrawal from study is voluntary; surgery is standard care independent of participation. |
| Methods: participants, interventions, outcomes | 11c | Adherence strategies. Strategies to improve adherence to intervention protocols and monitoring procedures. | Recruitment and Informed Consent; Study Procedures; Data-quality and Validity Rules | Standardized verbal/written instructions, home-based 5-night measurement windows, device return procedures, and predefined validity criteria are described. |
| Methods: participants, interventions, outcomes | 11d | Relevant concomitant care. Relevant concomitant care and interventions permitted or prohibited. | Study Population; Study Procedures; Secondary Outcomes | Standard care continues. Chronic sedatives/sleep aids and treated or high-suspicion OSA requiring evaluation/treatment are exclusion criteria; medication use is recorded. |
| Methods: participants, interventions, outcomes | 12 | Outcomes. Primary, secondary, and other outcomes, including specific measurement variables, metrics, methods, and time points. | Table 2; Outcomes | The primary Garmin Sleep Score outcome and secondary sleep, PRV, questionnaire, quality-of-life, and weight outcomes are defined. |
| Methods: participants, interventions, outcomes | 13 | Participant timeline. Time schedule of enrollment, interventions/exposures, assessments, and visits. | Participant Timeline; Figure 1 | SPIRIT schedule figure presents screening/enrollment, baseline, surgery, 1-year follow-up, and analysis. |
| Methods: participants, interventions, outcomes | 14 | Sample size. Estimated number of participants and how it was determined. | Sample Size | Target n=50. The paired-means calculation is a pragmatic design approximation; nightly mixed-effects modeling remains the primary analysis. |
| Methods: participants, interventions, outcomes | 15 | Recruitment. Strategies for achieving adequate participant enrollment. | Recruitment and Informed Consent; Study Status | Recruitment through standard metabolic surgery pathway and group sessions at the Obesity Center; 12-month recruitment planned. |
| Methods: assignment of interventions | 16a | Allocation: sequence generation. Method of generating allocation sequence. | N/A | Not applicable: observational cohort without randomization or allocation. |
| Methods: assignment of interventions | 16b | Allocation concealment mechanism. Mechanism of implementing allocation sequence. | N/A | Not applicable: no allocation sequence. |
| Methods: assignment of interventions | 16c | Implementation. Who generates allocation sequence, enrolls participants, and assigns interventions. | N/A; Recruitment and Informed Consent | Not applicable to allocation; participant enrollment procedures are described. |
| Methods: assignment of interventions | 17a | Blinding. Who will be blinded after assignment and how. | N/A | Not applicable: no randomized treatment assignment and no blinded intervention. |
| Methods: assignment of interventions | 17b | Emergency unblinding. Procedure for revealing assignment if needed. | N/A | Not applicable: no blinding. |
| Methods: data collection, management, analysis | 18a | Data collection methods. Plans for assessment and collection of outcome, baseline, and other data. | Study Procedures; Measures and Devices; Data-quality and Validity Rules; Outcomes | Devices, questionnaires, diaries, version and update recording, weekday/weekend classification, valid-night rules, and the calculation of night-to-night variability are described. |
| Methods: data collection, management, analysis | 18b | Plans to promote participant retention. Plans to promote participant retention and complete follow-up. | Study Procedures; Study Status | Home measurement and alignment with routine visits reduce burden and support follow-up completion. |
| Methods: data collection, management, analysis | 19 | Data management. Plans for data entry, coding, security, storage, and data quality. | Data Management and Privacy; Monitoring and Quality Assurance | Coded/pseudonymized data, separate reidentification key, ResearchManager, secure hospital systems, data-sharing restrictions, and monitoring are stated. |
| Methods: data collection, management, analysis | 20a | Statistical methods: outcomes. Statistical methods for analyzing primary and secondary outcomes. | Analytic Strategies | The primary mixed-effects model includes measurement period and night type; paired period-level analysis is a sensitivity analysis. |
| Methods: data collection, management, analysis | 20b | Statistical methods: additional analyses. Methods for additional analyses, including subgroup and adjusted analyses. | Analytic Strategies, Secondary and Exploratory Analyses | Exploratory analyses address weight loss, clinical factors, diary-device agreement, season, medication changes, and device-version changes where estimable. |
| Methods: data collection, management, analysis | 20c | Missing data. Definition of analysis population and methods for handling missing data. | Analytic Strategies, Missing Data and Outliers | No outcome imputation; questionnaire missingness handled by published rules; covariate imputation may be used; complete vs incomplete paired data compared descriptively. |
| Methods: monitoring | 21a | Data monitoring. Composition of data monitoring committee, role, reporting structure, independence. | Monitoring and Quality Assurance | Risk-based monitoring is planned. No DSMB/data monitoring committee is planned because the study is low risk and observational. |
| Methods: monitoring | 21b | Interim analyses and stopping guidelines. Description of interim analyses and stopping guidelines. | N/A; Monitoring and Quality Assurance | No interim efficacy analyses or formal stopping rules are planned because the study is observational and low risk. |
| Methods: monitoring | 22 | Harms. Plans for collecting, assessing, reporting, and managing adverse events or unintended effects. | Ethical Considerations and Trial Registration; Monitoring and Quality Assurance | Potential risks are mild skin irritation, discomfort, pressure marks, inconvenience, and altered behavior. Reporting procedures are addressed through GCP/MEC-U monitoring. |
| Ethics and dissemination | 23 | Research ethics approval. Plans for seeking ethics approval. | Protocol Information; Ethical Considerations and Trial Registration | Approved by MEC-U, reference R26.043, 3 July 2026. |
| Ethics and dissemination | 24 | Protocol amendments. Plans for communicating important protocol modifications. | Ethical Considerations and Trial Registration; Monitoring and Quality Assurance | Substantial amendments will follow MEC-U procedures and will be reflected in the study registration where applicable. |
| Ethics and dissemination | 25 | Consent or assent. Who will obtain informed consent and how. | Recruitment and Informed Consent; Ethical Considerations and Trial Registration | Written informed consent after one-week consideration period and individual discussion opportunity. |
| Ethics and dissemination | 26a | Confidentiality. How personal information will be collected, shared, and maintained to protect confidentiality. | Data Management and Privacy | Coded/pseudonymized data, separate key, secure systems, restricted access, and limited data transfer to SmartQare/Garmin are described. |
| Ethics and dissemination | 26b | Additional consent for biological specimens. Consent provisions for collecting and using biological specimens. | N/A | Not applicable: no biospecimens are collected or retained. |
| Ethics and dissemination | 27 | Declaration of interests. Financial and other competing interests for investigators. | Declarations; Conflicts of Interest | Financial and non-financial disclosures and device support from SmartQare are stated. |
| Ethics and dissemination | 28 | Access to data. Who will have access to the final trial dataset and disclosure of contractual limits. | Data Management and Privacy; Availability of Data and Materials | Authorized research team access; SmartQare receives only technical viQtor data/analytical data as described; no reidentification key. |
| Ethics and dissemination | 29 | Ancillary and post-trial care. Provisions for ancillary/post-trial care and compensation for trial-related harm. | Ethical Considerations and Trial Registration | No study treatment is assigned. Standard clinical care continues independently of participation, and no study-specific post-trial care is required. |
| Ethics and dissemination | 30 | Dissemination policy. Plans for communicating results to participants, healthcare professionals, public, and publication plans. | Study Status; Conclusions | Results will be disseminated through peer-reviewed publication and scientific communication after study completion. |
| Appendices | 31a | Informed consent materials. Model consent form and related documentation. | Recruitment and Informed Consent; approved MEC-U study documents | Consent procedure is described in the manuscript; participant information/consent form exists in approved MEC-U dossier and can be supplied if requested. |
| Appendices | 31b | Biological specimens. Plans for collection, laboratory evaluation, and storage of biological specimens. | N/A | Not applicable: no biological specimens are collected. |
| Appendices | 32 | Model data collection forms. Case report forms, questionnaires, diaries, and other data collection forms. | Study Procedures; approved study forms and questionnaires | The diary, questionnaires, EDC forms, and device instructions are maintained in the approved study documentation. |
| Appendices | 33 | Supplementary material. Other relevant documents, such as full protocol, statistical analysis plan, and participant materials. | Supplementary Material; Figure 1 | The completed SPIRIT 2013 checklist is submitted as Supplementary Appendix 1; the SPIRIT schedule is Figure 1 in the manuscript. |

**Reference:** Chan A-W, Tetzlaff JM, Altman DG, et al. SPIRIT 2013 statement: defining standard protocol items for clinical trials. Ann Intern Med. 2013;158(3):200-207. doi:10.7326/0003-4819-158-3-201302050-00583
